# Supplementary material for: Two-component nematic superconductivity in 4Hb-TaS2
Source: Nat Commun. 2024 Jan 27;15:824. doi: 10.1038/s41467-024-45169-3 (PMC10821864; doi:10.1038/s41467-024-45169-3)
Supplement: Supplementary file 1 — Supplementary Information [file 41467_2024_45169_MOESM1_ESM.pdf]

Supplementary Information for: Two-Component Nematic Superconductivity in $4Hb$-TaS_2

I. Silber,[1] S. Mathimalar,[2] I. Mangel,[3] A.K. Nayak,[2] O. Green,[1] N. Avraham,[2] H. Beidenkopf,[2] I. Feldman,[3] A. Kanigel,[3] A. Klein,[4,5] M. Goldstein,[1] A. Banerjee,[6] E. Sela,[1] and Y. Dagan[1]

[1] *School of Physics and Astronomy, Tel - Aviv University, Tel Aviv, 69978, Israel*
[2] *Department of Condensed Matter Physics, Weizmann Institute of Science, Rehovot, Israel*
[3] *Physics Department, Technion-Israel Institute of Technology, Haifa 32000, Israel*
[4] *Department of Physics, Faculty of Natural Sciences, Ariel University, Ariel 40700, Israel*
[5] *Department of Chemical Physics, The Weizmann Institute of Science, Rehovot 76100, Israel*
[6] *Department of Physics, Ben-Gurion University of the Negev, Beer-Sheva 84105, Israel*

Contents

S1. Hall data of $4Hb$-TaS_2 2

S2. Control Experiments 3

S3. Proposed Origin of the Stripes 5

S4. Additional critical field data 6

S5. Averaging procedure of the vortex core 7

S6. Anisotropic Ginzburg–Landau theory with an in-plane field 8
- I. Incorporation of the stripe modulation via mass modulation 8
- II. Details of the numerical calculation 10

S7. Chiral to nematic transition 11

S8. Specific heat from the Ginzburg Landau theory 13
- I. Single component order parameter theory 13
- II. Two component order parameter theory 14
- III. Comparison to experiments 15
- IV. Modification to the Free energy $-$ B_z terms 16
- V. Minimization of the free energy, $\lambda_\eta \ll c_\eta \lesssim b_\eta$ 17

S9. Supplementary References 19

S1. Hall data of 4Hb-TaS_2

The charge density wave transition in 4Hb-TaS_2 changes the carrier density dramatically. A careful measurement of the temperature dependence of the hall number reveals a decrease by a factor of five across the CDW transition as can be seen in Fig. S1. The Hall number is not simply related to the actual carriers density in TaS_2 polymorphs [1, 2] due to the complicated Fermi surfaces [3]. However, we interpret the dramatic change in the Hall number as a result of a Fermi surface reconstruction at the CDW transition that gaps major parts of Fermi surface.

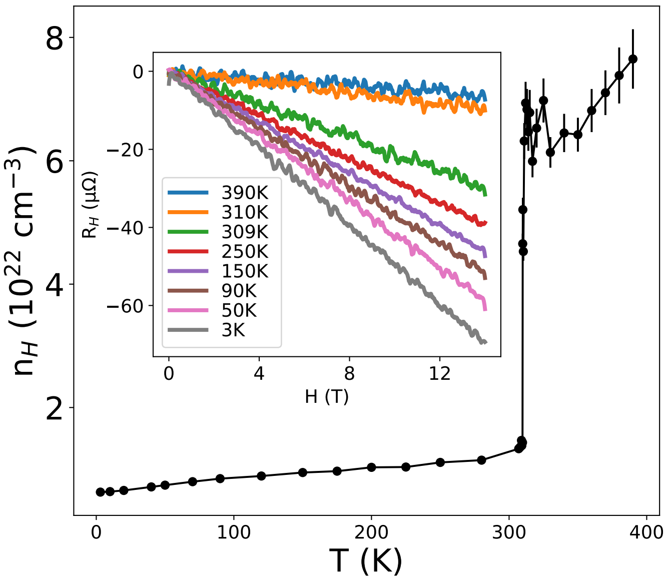

FIG. S1. The extracted Hall number as a function of temperature in a single crystal of 4Hb-TaS_2. Inset: raw measurements of the Hall resistance as a function of the field at various temperatures. Notice the rapid change of the slope by changing the temperature from T = 309 K to T = 310 K. Error bars mark the standard deviation of the linear fits, taking into account measurement uncertainties.

S2. Control Experiments

Our main result (Fig. 3) is a two-fold symmetric modulation of the critical field when the magnetic field is applied in the basal plane of the 4Hb-TaS_2 crystal. This effect is correlated with the stripe modulations seen on the microscopic scale. In this section we exclude three possible spurious origins for the modulations in H_{c2}, namely: current direction, sample misalignment and a wiggle of the plane of rotation.

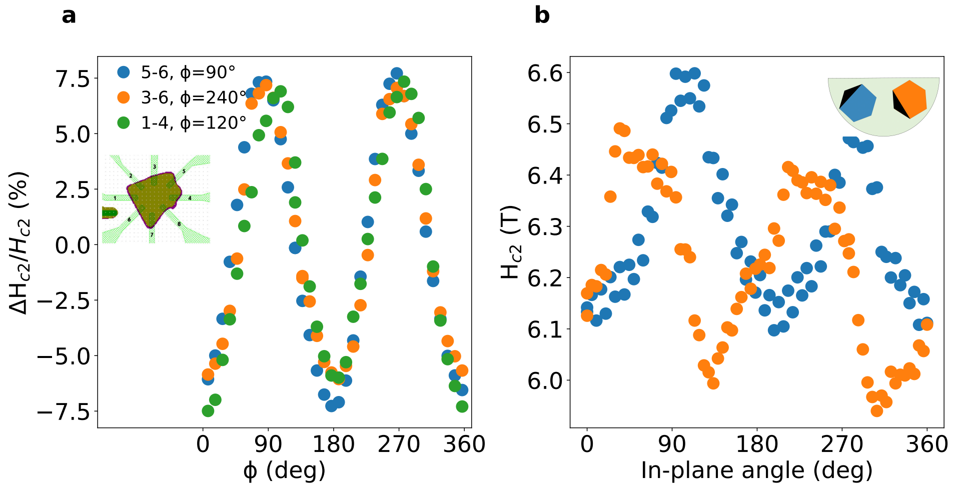

FIG. S2. Overview of control experiments using a one-axis rotator. (a) The critical field modulation at different contacts of the flake sample for various current directions depicted in Fig. 3a. Clearly, the current contacts has a negligible effect on the two-fold modulation. Inset: E-beam design of the eight gold contacts to the flake. (b) Critical field at $T = 2$ K as a function of the probe angle (the $\phi = 0$ direction is arbitrary here) for two cleaves of the same crystal. The two cleaved samples were co-mounted in a relative angle of about 90°. As expected from an intrinsic effect, the minimal critical field is shifted by a similar angle. The minimum critical field in both cases occurs when the field is applied along a crystal axes. The data in (b) rule out a presumed oscillating out of plane field component. The current in the blue curve is applied at $\phi = 170°$ and in the orange curve at $\phi = 70°$. Inset: a cartoon displaying how the two different cleaves are co-mounted on the rotating platform with a relative angle of $\simeq 90°$. The color of the illustrated cleave corresponds to the color of the data points in the main panel.

Current direction. The current direction might in general affect the critical field. To show that this is not an important effect in our data we performed an experiment in which the magnitude of the magnetic field was set to the middle of the superconducting transition. We then rotated the field in the basal plane, for four different applied current directions of the single crystal sample. We notice that the angular dependence is unchanged with the applied current direction. A finite resistance appears in directions where the critical field is the smallest independent of current direction, as can be seen at Fig. 3c. Similarly, the critical field was measured at various pairs of contacts in the flake sample. The two fold modulation is unchanged by the different contact configuration (see Fig. S2a).

Sample misalignment. A constant out-of-plane misalignment of an angle θ_0 will simply reduce that critical field by a constant factor, and will not change the in-plane angular dependence. If we assume that the entire difference in the critical fields is due to an out-of-plane components, we obtain $\theta_0 = 1.5°$. We also note that the minimal critical field of 4.3 T is very large for $T = 2.1$ K (see [3]), meaning the magnetic field is well aligned in the basal plane. We conclude that in our experiment the maximal misalignment is small ($< 1.5°$), and it is not the source of the observed effects.

Sample rotation wiggle. A more subtle error could be that the out-of-plane field component changes as we rotate the sample in the basal plane. This error could arise from a possibly misaligned rotation plane. We exclude such effect by performing control experiments. The two-fold symmetry of the critical field was reproduced for six different samples, in a total of four different rotation probes. In all of the samples the minimal critical field occurred when the critical field was pointed along the crystal's a-axis. Thus we conclude that the effect is due to the sample properties.

Secondly, we performed a control experiment in which two cleaves of the same crystal were co-mounted in an intentional 90° in-plane offset. If the variation of the critical field was only due to a presumed wobble in the rotation, we would expect that the critical field will be minimal for the two samples at the same angles. However, we observed that the critical field minimum is rotated by 90°, meaning that the field variation is related only to the crystal and not to the (common) rotation platform (Fig. S2b). We note that here the critical field is defined as the field at which

the resistance is 90% of the normal state resistance.

Finally, we performed a control experiment using a two-axis rotator. We carefully calibrated the precise in-plane angle θ_{plane} value for nine ϕ angles spanning the 180 degrees range available in the rotator. This calibration was achieved by observing the distinct in-plane dip in the resistance at the superconducting phase, as illustrated in Fig. S3a. By utilizing this calibration information (depicted in Fig. S3b), we were able to factor in the minuscule out-of-plane wobbling of the sample during the in-plane angle rotation. Consequently, the out-of-plane angle θ adjusted in conjunction with ϕ, using a linear interpolation of the calibration points, to zero the out-of-plane component during the rotation of the angle ϕ. Clearly, a two-fold oscillations in the resistance is measured for fields around the critical field. At zero field and in the normal state the resistance is independent of the in-plane angle, see Fig S3c.

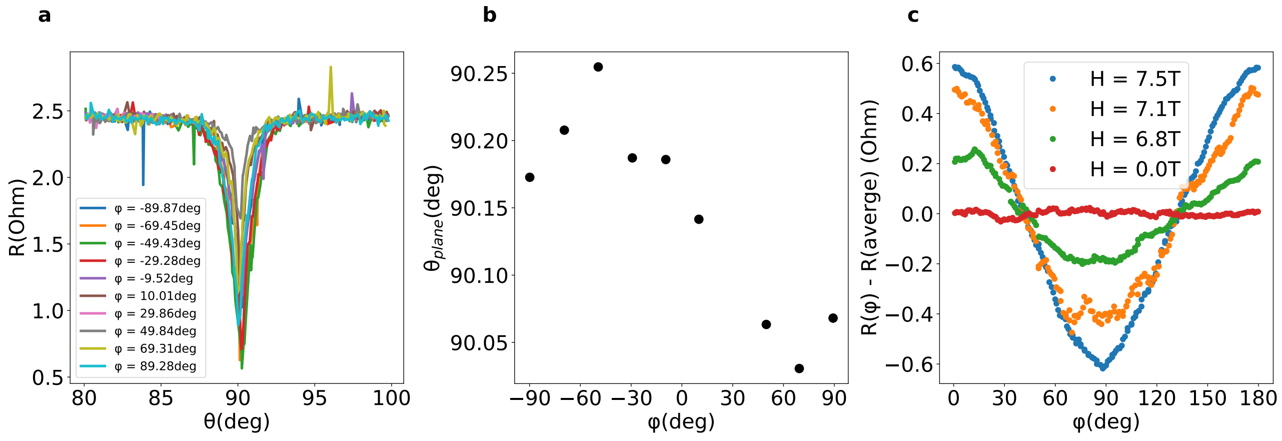

FIG. S3. Control experiments using a two-axis rotator. (a) Raw data of resistance as a function of out-of-plane angle, at $T = 1.7$ K and $H = 7.5$ T, at various probe angles (the $\phi = 0$ direction is arbitrary here) displaying a clear minimum when the field is in the plane of the sample. (b) The extracted in-plane angle as a function of the rotator angle. A minuscule change in the alignment is found for the entire range. (c) The resistance, shifted by its average, as a function of the in-plane angle at various fields at $T = 1.7$ K. Here, the out-of-plane angle was adjusted for every angle ϕ, using the calibration curve in (b). Clearly, two-fold modulation of the resistance is observed. Here $\phi = 0$ was adjusted to the maximum resistance, similar to the other flake samples in the main text. The current is applied at $\phi = 80°$.

S3. Proposed Origin of the Stripes

We interpret the stripe pattern as a result of the layer mismatch in 4Hb-TaS_2. Let us consider the geometrical aspects of the 4Hb-TaS_2 layers. The 1H and 1T layers have different in-plane lattice parameters with about 1.5% mismatch [4] and different symmetries [5]. Without any special symmetry considerations, this mismatch will either relax isotropically or lead to a two dimensional Moiré pattern. However, at the charge ordered states the 1T and 1H layers form different charge density waves, leading to a non isotropic mismatch. Specifically, the CDW pattern in the 1H layer is parallel to one of the crystal axes [6]. In this special direction the strain is maximal and the charge moves to minimize the CDW-induced mismatch.

This uniaxial contraction may lead to a one dimensional Moiré-like pattern parallel to the CDW pattern, with the expected periodicity of 19 nm due to the 1T and 1H lattice 1.5% mismatch [7]. The strong electron-phonon coupling in 4Hb-TaS_2 couples the electronic degrees of freedom to the lattice, resulting in a Moiré-like pattern of the local density of states, as seen in Fig. 1c. We speculate that scarce regions with no stripes are due to some local strain relaxation, as the stripes tend to deform close to a dislocation, as seen in Fig. S4.

An alternative explanation is that the stripes are a manifestation of a triclinic charge density wave, that is seen in the 1T-TaS_2 polymorph in very narrow temprature range [8].

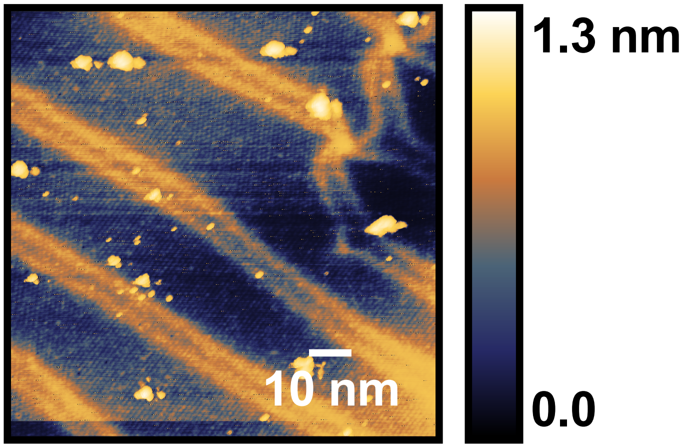

FIG. S4. Stripe pattern deformation around a dislocation. Local topography measured by an STM in a region with a dislocation (top right of the image), clearly showing the bending of the stripes next to it.

S4. Additional critical field data

At low temperature, T = 0.4 K, a much higher field, 16-18 T, is needed in order to suppress superconductivity in 4Hb-TaS_2. We performed measurements on a single crystal sample using a two-axis rotator in the National High Magnetic Field Laboratory (NHMFL) facility. We have found a much weaker anisotropy of H_{c2}, hardly resolved from the data, as seen in Fig. S5.

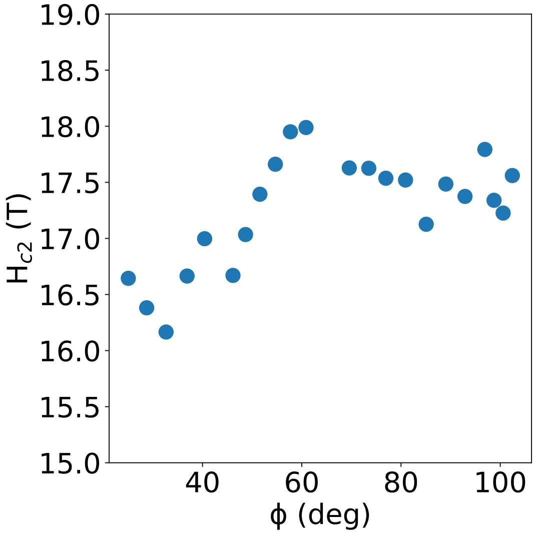

FIG. S5. Weaker critical field anisotropy at low temperatures. The critical field H_{c2} as a function of the in-plane angle at T=0.4 K.The current is applied at $\phi = 0°$.

The low temperature data is in stark contrast to the higher temperature data, in which the change in the critical field can be observes directly in the raw data (Fig. S6).

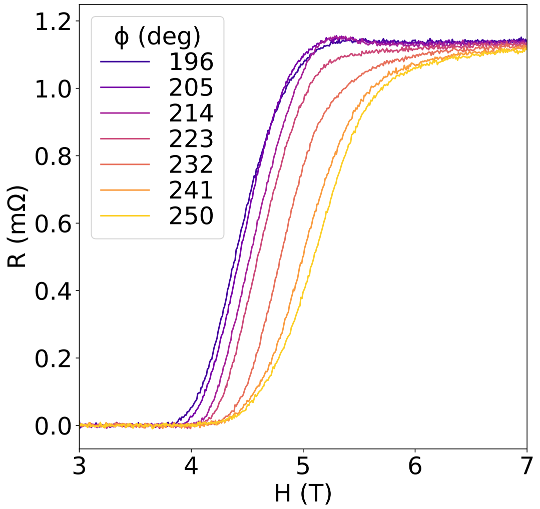

FIG. S6. Raw field scan to deduce the critical field. The magnetic field scans were performed at different in-plane angles while keeping a constant temperature. The critical field is clearly changing as a function of the in-plane angle. We show only a quarter cycle to avoid over-lapping of the measurements. The current is applied at $\phi = 20°$.

S5. Averaging procedure of the vortex core

We image the vortex core by scanning a large field of view in the presence of an out-of-plane field. The energy is set to match the Fermi energy (zero bias). This yields the raw conductance data in Fig. S7a. The raw image is smoothed spatially by applying a two dimensional Gaussian filter, taking the standard deviation to be 2. We select five vortices that are fully covered in the field of view and without any clear drifts, as seen in Fig. S7b. Those vortices are averaged to yield the figure presented in Fig. 1e.

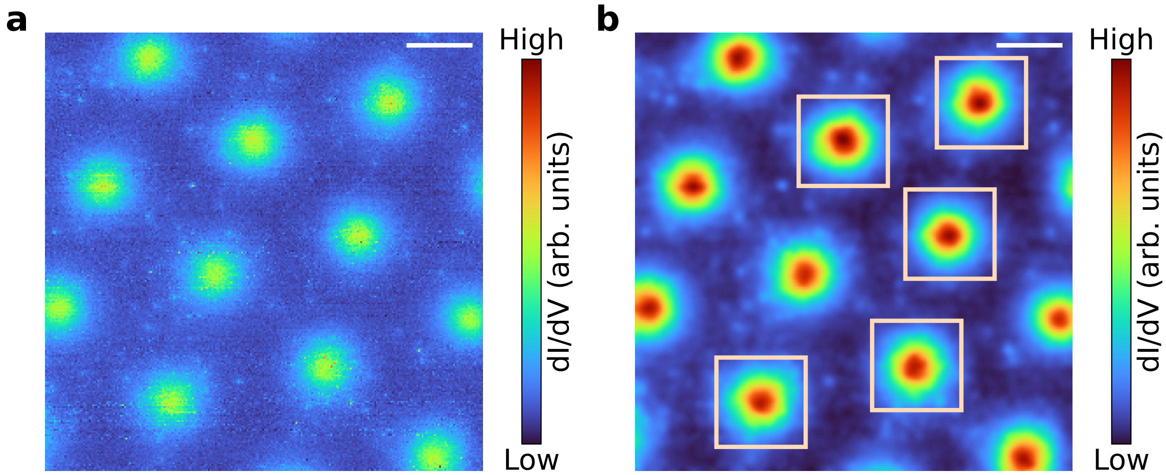

FIG. S7. Imaging a vortex core. (a) The raw measurements taken at zero bias in the presence of an out-of-plane field of 100 mT at T = 0.38 K. (b) Gaussian smoothing of the raw measurements presented in (a). The five vortices enclosed by boxes were used to calculate the averaged vortex profile shown in Fig. 1e. In both images the scale bar corresponds to 75 nm.

S6. Anisotropic Ginzburg–Landau theory with an in-plane field

Our starting point is the linearized GL equation

$$\sum_{i=x,y,z} \frac{1}{2m_i}(-i\hbar\nabla_i - 2eA_i)^2\psi + \alpha\psi = 0, \tag{1}$$

where the masses are related to the coherence length, $m_x = m_y \equiv m_{xy} = \frac{\hbar^2}{2\xi_{xy}^2|\alpha|}$, and $m_z = \frac{\hbar^2}{2\xi_z^2|\alpha|}$. Treating $H_0 = \sum_i \frac{1}{2m_i}(-i\hbar\nabla_i - 2eA_i)^2$ as the Hamiltonian of a particle in a magnetic field with eigenvalues $\{E\}$, the critical field is obtained from the minimal energy solution, $|\alpha| = \min\{E\}$. For the homogeneous case with a magnetic field in the xy plane we have eigenstates $|k_\parallel, k_\perp, n\rangle$ (k parallel or perpendicular to the magnetic field B in the plane) with energy

$$E = \hbar\omega(n + 1/2) + \frac{\hbar^2 k_\parallel^2}{2m_{xy}} + \frac{\hbar^2 k_z^2}{2m_z}, \tag{2}$$

where, $\omega = \frac{2eB}{\sqrt{m_{xy}m_z}}$ so that $\min\{E\} = \frac{\hbar\omega}{2}$, and

$$H_c = \frac{|\alpha|\sqrt{m_{xy}m_z}}{\hbar e} = \frac{\Phi_0}{\xi_{xy}\xi_z}, \tag{3}$$

with $\Phi_0 = \hbar/2e$. For $B \parallel z$ we have $H_{c,z} = \frac{\Phi_0}{\xi_{xy}^2}$. Since $\xi_{xy} \gg \xi_z$ the in-plane critical field is larger than the perpendicular critical field, $\frac{H_{C,xy}}{H_{C,z}} = \frac{\xi_{xy}}{\xi_z} \gg 1$.

I. Incorporation of the stripe modulation via mass modulation

Here the anisotropy of the critical field is attributed to a modulation of the perpendicular mass m_z in a GL theory. The stripes are incorporated by adding a periodic modulation to m_z,

$$m_z(x) = m_z + \delta m \cos\frac{2\pi x}{a}. \tag{4}$$

Here a is the distance between the stripes, and we set the x direction to run perpendicular to the stripes. We treat this modulation as a small perturbation, $\delta m \ll m_z$. The Hamiltonian used to obtain the critical field is $H = H_0 + V$, where

$$V = -\frac{\delta m}{4m_z^2} p_z^2 (e^{i\frac{2\pi x}{a}} + e^{-i\frac{2\pi x}{a}}) + V^{(2)} + O(\delta m^3), \tag{5}$$

Here $V^{(2)} = \frac{p_z^2}{2m_z}\left(\frac{\delta m}{m_z}\right)^2 \cos^2(2\pi x/a)$ and p_z is the momentum operator along the z direction. We next compute the correction δE to the ground state energy. The relative correction to the critical field is

$$\frac{\delta H_c}{H_c} = -2\frac{\delta E}{\hbar\omega}. \tag{6}$$

B ∥ z. For perpendicular field the screening currents flow in the xy plane and are unaffected by the periodic modulation in m_z. Thus, $H_{c,z}$ is unaffected by δm. Next we consider an in-plane field, $\mathbf{B} = B(\cos(\theta)\hat{x} + \sin(\theta)\hat{y})$.

B ⊥ stripes ($\theta = 0$). We have set the x direction to run perpendicular to the stripes so that in the present case $B \parallel \hat{x}$, and the cyclotron motion takes place in the yz plane. The perturbation acts nondiagonally on the momentum $|k_\parallel\rangle \to |k_\parallel \pm \frac{2\pi}{a}\rangle$, increasing the eigenvalue of H_0 by $E_{BZ} = \frac{\hbar^2}{2m_{xy}}\left(\frac{2\pi}{a}\right)^2$. We define the ratio

$$W = \frac{E_{BZ}}{\hbar\omega} = \left(\frac{\pi\ell_B}{a}\right)^2 \frac{\xi_{xy}}{\xi_z} \gg 1, \tag{7}$$

where $\ell_B = \sqrt{\frac{\hbar}{eB}}$ is the magnetic length. Then, up to second order, using $p_z = -i\sqrt{\frac{m_z\hbar\omega}{2}}(a - a^\dagger)$ we have $\frac{\delta E}{\hbar\omega} =$

$-\frac{\delta m^2}{32m_z^2}\frac{1}{W}\left(1+\frac{2}{1+2W^{-1}}\right)+\frac{\delta m^2}{8m_z^2}$. The two terms inside the parenthesis correspond to a virtual transition to $k_\parallel = \pm 2\pi/a$ and either $n' = 0$ or $n' = 2$, respectively. Here n, n' denote eigenvalues of $a^\dagger a$. The last term stems from first order perturbation theory in $V^{(2)}$. This quadratic dependence on the mass modulation δm is confirmed in Fig. S8(a) via a comparison to a numerical solution. From Eq. (6), the correction to the critical field is

$$\frac{\delta H_c^\perp}{H_c} = \frac{\delta m^2}{16m_z^2}\frac{1}{W}\left(1+\frac{2}{1+2W^{-1}}\right) - \frac{\delta m^2}{4m_z^2}. \tag{8}$$

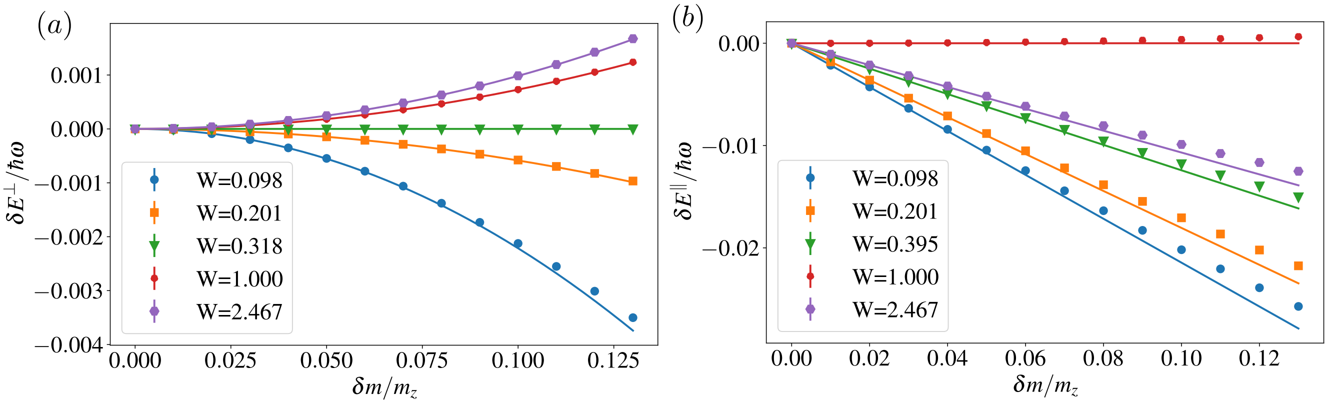

FIG. S8. Correction to the lowest energy eigenvalue with increasing mass variation $\delta m/m_z$ presented for different W. The points are the results from the numerical calculation whereas the lines are the perturbative results. (a) $\theta = 0$ that is magnetic field perpendicular to the stripe direction. The perturbative calculation predicts the correction to be second order in $\delta m/m_z$, which is confirmed by the numerical calculations. In this case, the correction is expected to change the sign at $W = \frac{1}{8}(-5+\sqrt{57})$, which is also obtained from the numerics. (b) $\theta = \pi/2$, i.e., magnetic field parallel to the stripe direction. Here the perturbation calculation predicts a first-order correction to the energy eigenvalue, which is confirmed by the numerical calculations. The first-order correction is expected to vanish for $W = 1$.

B ∥ stripes ($\theta = \pi/2$). In this case the cyclotron motion lies in the xz plane, and the unperturbed Hamiltonian is

$$H_0 = \frac{(\hbar k_x - 2eBz)^2 + (\hbar k_y)^2}{2m_{xy}} + \frac{(-i\hbar\nabla_z)^2}{2m_z}. \tag{9}$$

Adding the perturbation V, each momentum kick $k_x \to k_x \pm 2\pi/a$ acts as a shift operator $e^{\mp i p_z \Delta z/\hbar}$ on the oscillator, where $\Delta z = \frac{\ell_B^2}{2}\frac{2\pi}{a}$. Notice that in this case H_0 displays the Landau level degeneracy with respect to k_x. Performing degenerate first order perturbation theory, we obtain a hopping amplitude t between states differing by $\Delta k_x = \pm 2\pi/a$ and having exclusively $n = 0$,

$$\begin{aligned} t &= \langle k_x \pm 2\pi/a, k_y, n=0|V|k_x, k_y, n=0\rangle \\ &= -\frac{\delta m}{4m_z^2}\langle 0|e^{\mp i p_z \Delta z/\hbar} p_z^2|0\rangle. \end{aligned} \tag{10}$$

This is justified in the perturbative regime $t \ll \hbar\omega$, so that higher n will appear only in second order as $t^2/(\hbar\omega)$. Using the Harmonic oscillator result $\langle n|e^{\alpha a^\dagger - \alpha^* a}|0\rangle = \sqrt{\frac{1}{n!}}\alpha^n e^{-|\alpha|^2/2} \equiv f_n(\alpha)$, we have $\langle n|e^{\mp i p_z\Delta z/\hbar}\frac{p_z^2}{m_z\hbar\omega/2}|0\rangle = -\partial_\alpha^2 f_n(\alpha = \pm\sqrt{W})$. For $n = 0$, the correction to the lowest energy is negative and given by $\frac{\delta E}{\hbar\omega} = -\frac{2|t|}{\hbar\omega} = -\frac{\delta m}{2m_z}|W-1|e^{-\frac{W}{2}}$. This linear correction is confirmed via a comparison to a numerical simulation in Fig. S8(b). Thus the correction to the critical field is

$$\frac{\delta H_c^\parallel}{H_c} = \frac{\delta m}{m_z}|W-1|e^{-\frac{W}{2}}. \tag{11}$$

Thus, this model with a varying mass along z predicts an anisotropic H_{c2}. To find the angle at which H_{c2} is maximal we compare the results for the critical field perpendicular or parallel to the stripes, Eqs. (8) and (11). We note that these equations carry a different dependence on W, determined by the typical distance between stripes. Rather than

plugging in a specific value of W, we note that there are sizable variations of the stripe separations. We thus drop the W dependencies of Eqs. (8) and (11) as coefficients of order unity. Thus, for small δm, the first order correction for field along the stripes is dominant, leading to a relative enhancement of the critical field, $\delta H_c^{\parallel} \gg \delta H_c^{\perp}$.

This conclusion applies beyond the perturbative regime, and also with not strictly periodic modulations. To verify this we performed a numerical solution of the effective Schrödinger equation away from the perturbative regime. As seen in Fig. S9, for generic values of W and $\delta m/m_z$ we have $\delta H_c^{\parallel} > \delta H_c^{\perp}$. We also present results from for non-periodic m_z modulations in Fig. S9(d). We see that the same sign of the anisotropy (maximal critical field along stripes) persists away from the periodic model as well.

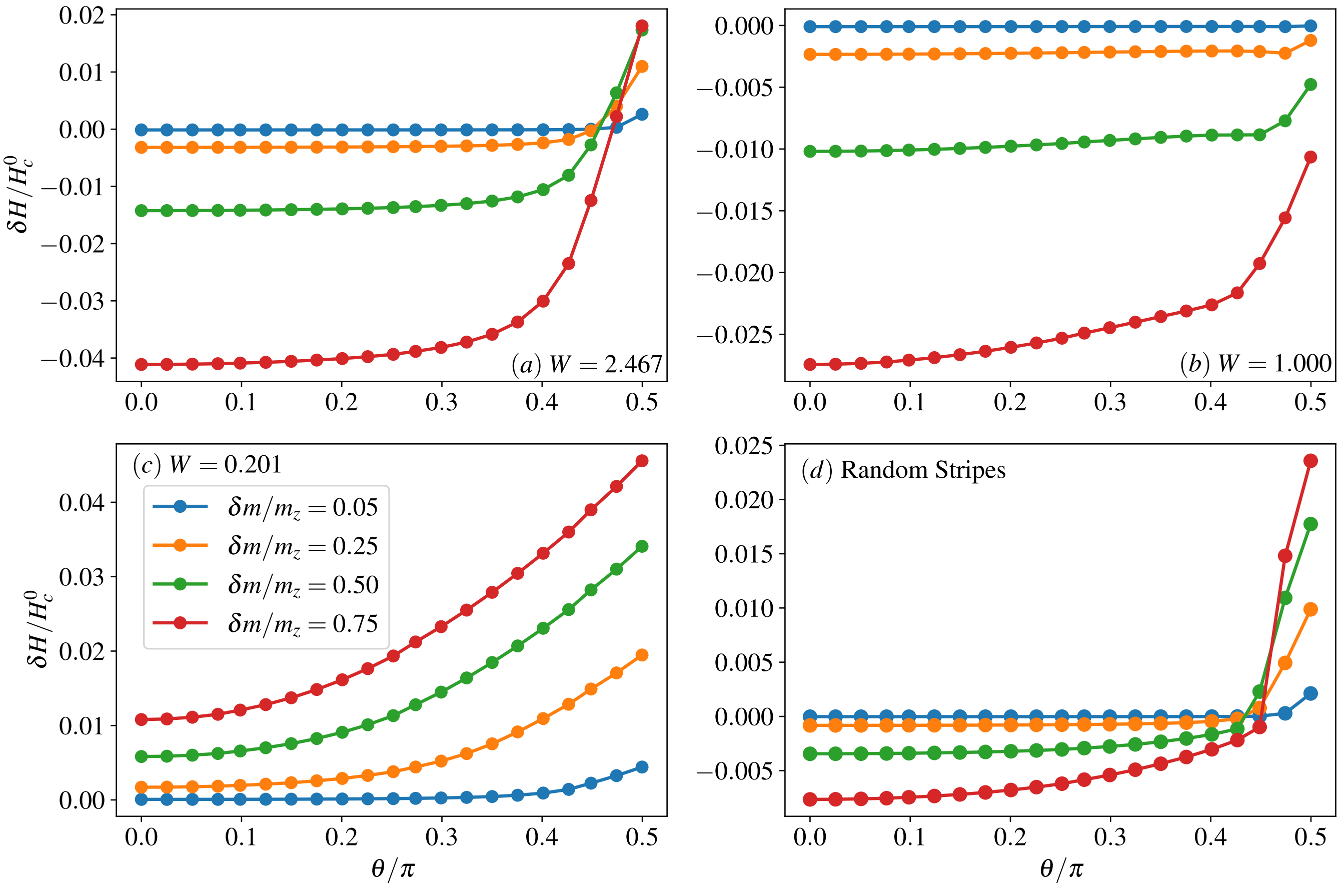

FIG. S9. Critical field versus θ ($\theta = \pi/2$ corresponds to field parallel to the stripes) for various values of the mass modulation $\delta m/m_z = 0.05, 0.25, 0.5, 0.75$. (a,b,c): the calculation is done for various W withing the model with periodic modulations. (d): Here we consider non-periodic mass variations as described in the text, with parameters $n = 2$, $a_1 = \sqrt{5}, a_2 = \sqrt{11}$, $b_1 = \sqrt{7}$, and $b_2 = \sqrt{13}$.

To create non-periodic stripes, we used the sum of sine and cosine functions with the irrational periodicity, which generates a profile of non-periodic stripes. The mass is now given by $m_z(x) = m_z + \delta m \sum_n c_n \cos(q_n x) + d_n \sin(r_n x)$ where $q_n = 2\pi/a_n$ and $r_n = 2\pi/b_n$. We select irrational a_n and b_n, and c_d and d_n were chosen randomly from a uniform distribution $[0, 1]$. We normalize the c_n and d_n as $\sum_n c_n + d_n = 1$, so that the maximum variation of the stripes is given by δm. We plot the disorder averaged correction to the field for 15 independent configurations. In particularly, in Fig. S9(d) we use $n = 2$ and choose the following parameters: $a_1 = \sqrt{5}, a_2 = \sqrt{11}$ and $b_1 = \sqrt{7}, b_2 = \sqrt{13}$.

II. Details of the numerical calculation

We solve the Schrodinger equation numerically for the lowest eigenstates using a grid in real space. We discretize the space in each dimension from $[a, b]$ and use periodic boundary conditions. We choose the grid size uniformly, and

hence the spatial coordinates are given by

$$r_i = a + \frac{b-a}{N} i \tag{12}$$

where N is the number of grid points in each direction, and $i = 1 \cdots N$. The quantities that depend only on the coordinates are sampled on these grid points. The derivatives are approximated using the finite-difference approach [9]. We used a fourth-order approximation of the Laplacian operator. We diagonalized the Hamiltonian using the standard procedure and keep track of the lowest eigenstates. To find the continuum limit we varied N from $N = 101$ and up to $N = 151$, and extrapolated the results to $N \to \infty$.

S7. Chiral to nematic transition

Let us consider the free energy of a two-component order parameter $\boldsymbol{\eta} = (\eta_x, \eta_y)^T$ [10, 11]:

$$F_\eta = r_\eta \boldsymbol{\eta}^* \cdot \boldsymbol{\eta} + b_\eta (\boldsymbol{\eta}^* \cdot \boldsymbol{\eta})^2 + c_\eta |\boldsymbol{\eta}^* \times \boldsymbol{\eta}|^2 + \kappa_1 \left((\boldsymbol{D}\eta_x)^* \cdot \boldsymbol{D}\eta_x + (\boldsymbol{D}\eta_y)^* \cdot \boldsymbol{D}\eta_y \right) + \kappa_2 \left(|\boldsymbol{D} \cdot \boldsymbol{\eta}|^2 - |\boldsymbol{D} \times \boldsymbol{\eta}|^2 \right). \tag{13}$$

Here $\boldsymbol{D} = -i\boldsymbol{\nabla} - 2e\boldsymbol{A}$ and $r_\eta \propto T - T_c^{(\eta)}$ sets the onset of this two component order parameter. We ignore for simplicity the interplay of η with a possible s-wave order parameter. The order parameter is parameterized as

$$\boldsymbol{\eta} = \eta \begin{pmatrix} \cos\alpha \\ \sin\alpha \; e^{i\gamma} \end{pmatrix}, \tag{14}$$

namely

$$\begin{aligned} \text{purely nematic}: \gamma = 0 \text{ or } \alpha = 0, \pi, \\ \text{purely chiral}: \alpha = \pi/4, \gamma = \pm\pi/2, \end{aligned} \tag{15}$$

see Fig. S10b.

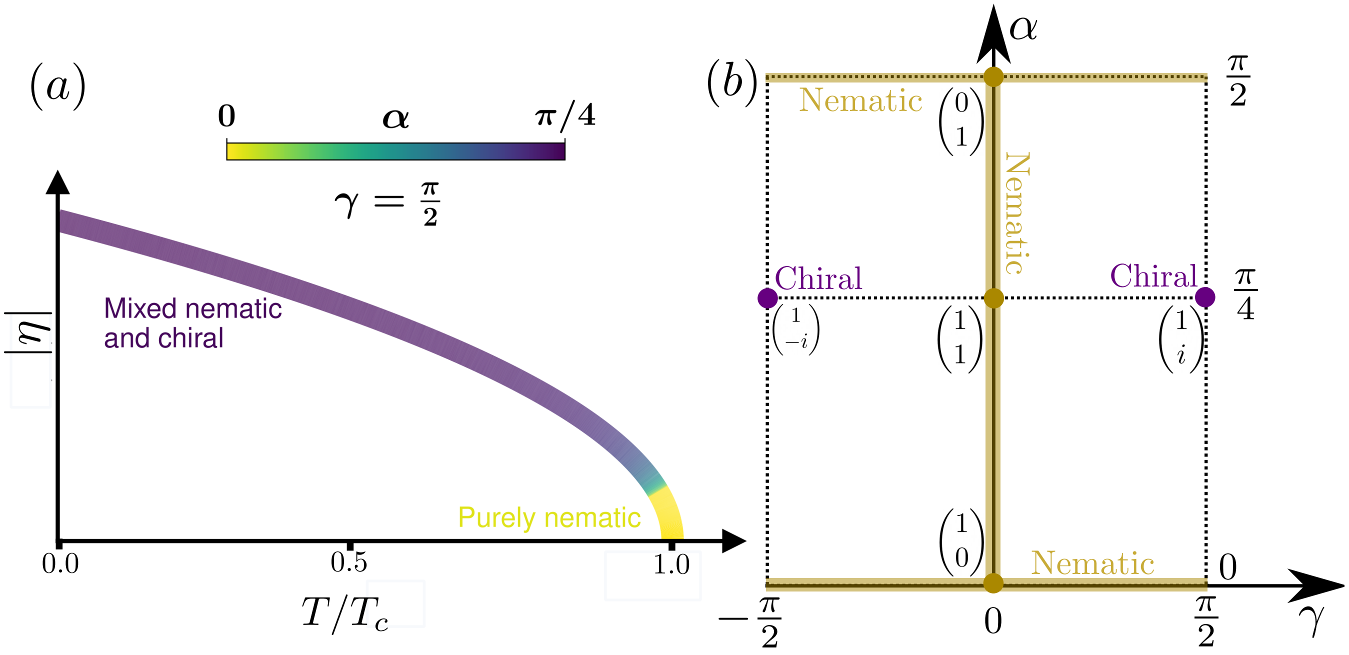

FIG. S10. The evolution of the two-component superconducting order parameter. (a) Schematic amplitude and character (color code) of the order parameter versus T, resulting from minimization of Eq. (17). (b) Chiral versus nematic parametrization of the order parameter using two angles (α, γ), see Eq. (14).

The term c_η selects chiral order for negative values, or nematic order for positive values. We assume $c_\eta < 0$ [3], which implies a tendency to a uniform chiral order induced at a temperature $T^* < T_c$, driven by the nonlinear coupling

which is negligible at $T = T_c$. In principle, there is another, quadratic, term that promotes chiral order in the presence of a magnetic field [10], of the form $iB\boldsymbol{\eta}\times\boldsymbol{\eta}^*$. Such a term would promote chiral order near vortex centers but not in the bulk of the material. We neglect this term here since it is (a) typically quite small and (b) is inconsistent with the disappearance of the H_{c2} anisotropy at low temperatures, see Fig. S5. In the presence of uniaxial strain we add the symmetry allowed term [10]

$$F_\epsilon = \lambda_\eta(\epsilon_{xx}-\epsilon_{yy})(\eta_x^*\eta_x - \eta_y\eta_y^*), \tag{16}$$

which favors nematic order.

To capture qualitatively the experimental features we apply the following procedure. We first ignore gradient terms, and for a given set of parameters r_η, b_η, c_η and $\lambda_\eta(\epsilon_{xx}-\epsilon_{yy})$ we solve for the degree of nematicity versus chirality, encoded in the pair (α,γ), as well as the amplitude η of the order parameter. This is obtained by minimizing the homogeneous free energy function

$$f_h(\eta,\alpha,\gamma) = r_\eta\eta^2 + b_\eta\eta^4 + c_\eta\eta^4\sin^2(2\alpha)\sin^2(\gamma) - \lambda_\eta(\epsilon_{xx}-\epsilon_{yy})\eta^2\cos(2\alpha). \tag{17}$$

While the $c_\eta < 0$ term favoring chirality is quartic in η, the strain term favoring nematicity is quadratic in η. Hence, for temperature slightly below T_c the system is nearly purely nematic, and gradually becomes chiral at low temperatures as depicted in the color code in Fig. S10a.

To describe this explicitly, we assume for simplicity that $b_\eta \gg c_\eta$, so that the amplitude of the order parameter is minimized independently, $\eta^2 = \frac{-r_\eta}{2b_\eta}$ for $r_\eta < 0$ and $\eta = 0$ otherwise, as displayed in Fig. S10a. Minimizing with respect to γ gives $\gamma = \pi/2$, so that we are bounded to the eastern vertical edge of the phase diagram in Fig. S10a. At $T = T_c$ ($r_\eta = 0$) we have a minimum of f_h at $\alpha = 0$. Minimizing with respect to α gives

$$\frac{c_\eta(-r_\eta)}{b_\eta\lambda(\epsilon_{xx}-\epsilon_{yy})}\cos(2\alpha) = -1. \tag{18}$$

This acquires a solution only below a temperature $T^* \le T_c$ where $\frac{c_\eta(-r_\eta)}{b_\eta\lambda(\epsilon_{xx}-\epsilon_{yy})} \ge 1$. Accordingly, as shown Fig. S10(a), the order parameter is purely nematic for $T^* < T < T_c$, and is mixed nematic and chiral below T^*. Deep in the superconducting phase it is maximally chiral.

Next, given α and γ, we allow a spatial dependence of the form $\boldsymbol{\eta} = \eta(x,y)(\cos\alpha, \sin\alpha e^{i\gamma})^T$. Substituting this into the gradients terms, they become $\kappa_1 F_{\kappa_1} + \kappa_2 F_{\kappa_2}$, where

$$\begin{aligned} F_{\kappa_1} &= |D_x\eta|^2 + |D_y\eta|^2, \\ F_{\kappa_2} &= \cos(2\alpha)(|D_x\eta|^2 - |D_y\eta|^2) + \sin(2\alpha)\cos\gamma(D_x\eta^* D_y\eta + D_y\eta^* D_x\eta). \end{aligned} \tag{19}$$

We can see that in the purely chiral case $F_{\kappa_2} = 0$, whereas in the purely nematic case F_{κ_2} is a simple mass anisotropy in the direction dictated by α, $F_{\kappa_2} = |D_{x'}\eta|^2 - |D_{y'}\eta|^2$ where $\begin{pmatrix} x' \\ y' \end{pmatrix} = \begin{pmatrix} \cos\alpha & \sin\alpha \\ -\sin\alpha & \cos\alpha \end{pmatrix}\begin{pmatrix} x \\ y \end{pmatrix}$. Thus the κ_2 term (denoted by J_4 in Ref. 10) is responsible for a possible anisotropy of either critical fields or of perpendicular vortices. This is dictated by a mass ratio

$$\frac{m_{y'}}{m_{x'}} = \frac{\kappa_1 + \frac{\kappa_2}{2}\sqrt{3+\cos(2\gamma)+2\cos(4\alpha)\sin^2\gamma}}{\kappa_1 - \frac{\kappa_2}{2}\sqrt{3+\cos(2\gamma)+2\cos(4\alpha)\sin^2\gamma}}. \tag{20}$$

In the fully chiral case we recover isotropy $\frac{m_{y'}}{m_{x'}} = 1$, while in the nematic case this becomes $\frac{m_{y'}}{m_{x'}} = \frac{\kappa_1+\kappa_2}{\kappa_1-\kappa_2}$.

We may now accommodate the experimental results within this model. When $T \ll T_c$ the order parameter is primarily chiral, $\alpha \cong \pi/4$, $\gamma = \pi/2$, so *isotropic vortices* will be observed. At higher temperatures, this model predicts an *anisotropic critical field*, which is dominated by the strain $\epsilon_{xx}-\epsilon_{yy}$. Whether the maximal critical field is parallel or perpendicular to the uniaxial strain depends on the signs of κ_2 and λ. It is expected that details of the microscopic model can yield either sign of the anisotropy of the in-plane H_{c2}.

Our model predicts that when the order parameter $|\eta|$ weak, nematicity is preferred. One might claim that at the center of the vortex core the vanishingly small order parameter is therefore nematic and we should expect anisotropic vortices for all temperatures. However, the STM measurement effectively probes a finite distance from the core, into the bulk, where the chiral nature of the order parameters is restored and we still expect that the shape of the vortex will represent the bulk order parameter.

Finally, we comment on the behavior of H_{c2} at $T \ll T_c$, and the suppression of the anisotropy we found at low temperatures. To obtain the behavior of H_{c2} at low T it is necessary to solve the Landau level problem for the in-plane

magnetic field, and then find the energy minimum by varying α and γ. Instead of this complicated procedure, we just show that the nematic part of the phase diagram is suppressed at low T. To this end, we estimate the instability of the chiral phase as a function of increasing magnetic field strength. In the chiral phase, the gap is isotropic, and at small magnetic field the gradient terms merely renormalize r_η,

$$r_\eta \to r_\eta + \frac{\hbar e H}{\sqrt{m_{xy} m_z}} = -|r_\eta| \left(1 - \frac{H}{H_c}\right), \tag{21}$$

see Eqs. (2) and (3). Plugging this back into the condition for the stability of the chiral phase, we find an estimate for the critical field strength

$$\frac{H^*}{H_c} \sim 1 - \frac{b_\eta \lambda(\epsilon_{xx} - \epsilon_{yy})}{c_\eta |r_\eta|}. \tag{22}$$

The nematic phase exists for $H^* < H < H_c$. Thus, the region of nematic superconductivity decreases sharply at low temperatures, where r_η is large.

S8. Specific heat from the Ginzburg Landau theory

We now turn to study the specific heat predicted by our theory. For simplicity, we neglect a possible s-wave contribution and study the effects solely caused by the two-component order parameter.

I. Single component order parameter theory

The GL theory for the homogeneous single component order parameter ψ is given by

$$\mathcal{F}_s = a_s (T - T_c) |\psi|^2 + b_s |\psi|^4. \tag{23}$$

At $T < T_c$ the coefficient of the quadratic term becomes negative and hence the order parameter condenses with $|\psi| = \sqrt{\frac{a_s(T_c - T)}{2b_s}}$, whereas for $T > T_c$ the order parameter vanishes, $\psi = 0$. Using Maxwell's relation we can calculate the entropy by

$$S = -\frac{\partial \mathcal{F}_s}{\partial T}. \tag{24}$$

When $T < T_c$, we thus obtain

$$S = \frac{a_s^2 (T - T_c)}{2b_s}. \tag{25}$$

The heat capacity of the superconductor is given by

$$C = T \frac{\partial S}{\partial T}. \tag{26}$$

Thus, the specific heat of the superconductor for $T < T_c$ is given by

$$C = \frac{a_s^2}{2b_s} T. \tag{27}$$

Note that the specific heat contribution due to the SC phase above T_c vanishes. Therefore, a jump of specific heat is expected at T_c which is given by

$$\Delta C(T_c) = \frac{a_s^2}{2b_s} T_c. \tag{28}$$

As expected jump in the specific heat for a single component order parameter is proportional to T_c.

II. Two component order parameter theory

Next, we evaluate the same for the two-component order parameter theory given by Eq. (17). The order parameter condenses for $T < T_c$ with

$$\eta = \sqrt{\frac{a_\eta (T_c - T)}{2b_\eta}}, \tag{29}$$

where we have defined $r_\eta = a_\eta(T - T_c)$. The specific heat in the ordered phase $T < T_c$ turns out to be

$$C = T\left(\frac{a_\eta^2}{2b_\eta} - \frac{a_\eta^2 c_\eta}{2b_\eta^2}\sin^2(2\alpha)\sin^2(\gamma)\right). \tag{30}$$

The specific heat in the normal phase vanishes due to the vanishing of the order parameter. Since our theory assumes that we are in a chiral state without the strain term, we must have $c_\eta < 0$. In addition, we found that $\gamma = \pi/2$, hence the above expression simplifies to

$$C = T\left(\frac{a_\eta^2}{2b_\eta} + \frac{a_\eta^2 |c_\eta|}{2b_\eta^2}\sin^2(2\alpha)\right). \tag{31}$$

We recall that near $T = T_c$ we are in a purely nematic phase, $\alpha = 0$. So the specific heat jump at T_c is given by

$$\Delta C = T_c \frac{a_\eta^2}{2b_\eta} \tag{32}$$

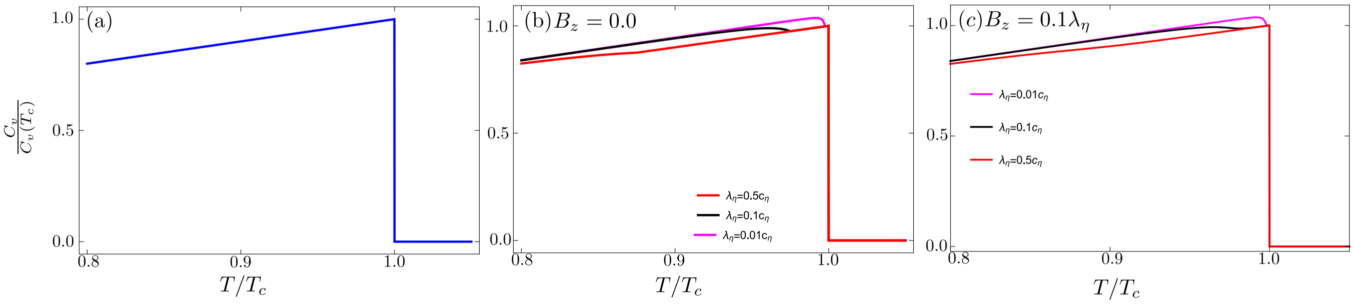

FIG. S11. Specific heat calculated from Ginzburg–Landau. (a) The variation of the specific heat as a function of temperature for a one-component order parameter theory. The specific heat exhibits a jump at the transition temperature T_c. (b) The same for the two-component order parameter theory. Here the specific heat also jumps at T_c. In addition, there is a kink of the specific heat at the crossover temperature T^*. At low strain, the kink in the specific heat becomes smaller. (c) The same for the two-component order parameter theory with the B_z term. Here the specific heat also has a similar jump at T_c, but the jump at T^* becomes less pronounced with increasing B_z.

The jump is the same as in the single component order parameter case. Thus, for a purely nematic phase one expects a specific heat jump proportional to T_c. We now consider the variation of the specific heat near the crossover to the mixed chiral-nematic phase at T^*. Here α is small and we can approximate $\sin(2\alpha) \approx 2\alpha$. The specific heat at T^* then becomes

$$C = T^* \frac{a_\eta^2}{2b_\eta}\left(1 + \frac{4|c_\eta|}{b_\eta}\alpha^2\right). \tag{33}$$

The specific heat increases and exhibit a kink at T^*.

We have plotted the superconducting contribution to the specific heat calculated within the GL formalism as a function of temperature in Fig. (S11), down to $T = 0.8T_c$. For single component order parameter theory shown in Fig. (S11a) we find the expected jump at T_c. Such a jump at $T = T_c$ is also present in the two-component order parameter theory as shown in Fig. (S11 b). In addition, there is a kink at T^* indicating the crossover from a purely

nematic to a mixed chiral-nematic state.

III. Comparison to experiments

The T^* in the GL theory is given by

$$T^* = T_c - \frac{b_\eta \lambda_\eta}{a_\eta |c_\eta|}. \tag{34}$$

We fix $a_\eta = 1 = b_\eta$. Our calculations are only valid in the regime of $\lambda_\eta \ll c_\eta \ll b_\eta$. Hence we set $c_\eta = 0.05 b_\eta$. T^* becomes well resolved for large strain λ_η in this scenario. To estimate different signatures of the transition let us focus primary experimental observations in these materials which we are trying to capture in the GL theory,

- Anisotropic H_{c2}, near the critical temperature persisting up to $T \sim 0.75T_c$.
- Isotropic vortices around $T \sim 0.15T_c$
- Specific heat shows only sharp fall at T_c, no signatures of T^* [3].

In the GL theory, the crossover temperature changes if the strain term is varied. The variation of the specific heat with the different strength of strain term λ_η is shown in Fig. S11b. Here we find that kink at T^* becomes smaller as the strain decreases. The specific heat becomes comparable to the experimental observations for $\lambda_\eta = 0.1c_\eta$. At this strain strength, $T^* \sim 0.9T_c$, which is difficult to resolve in the experiments.

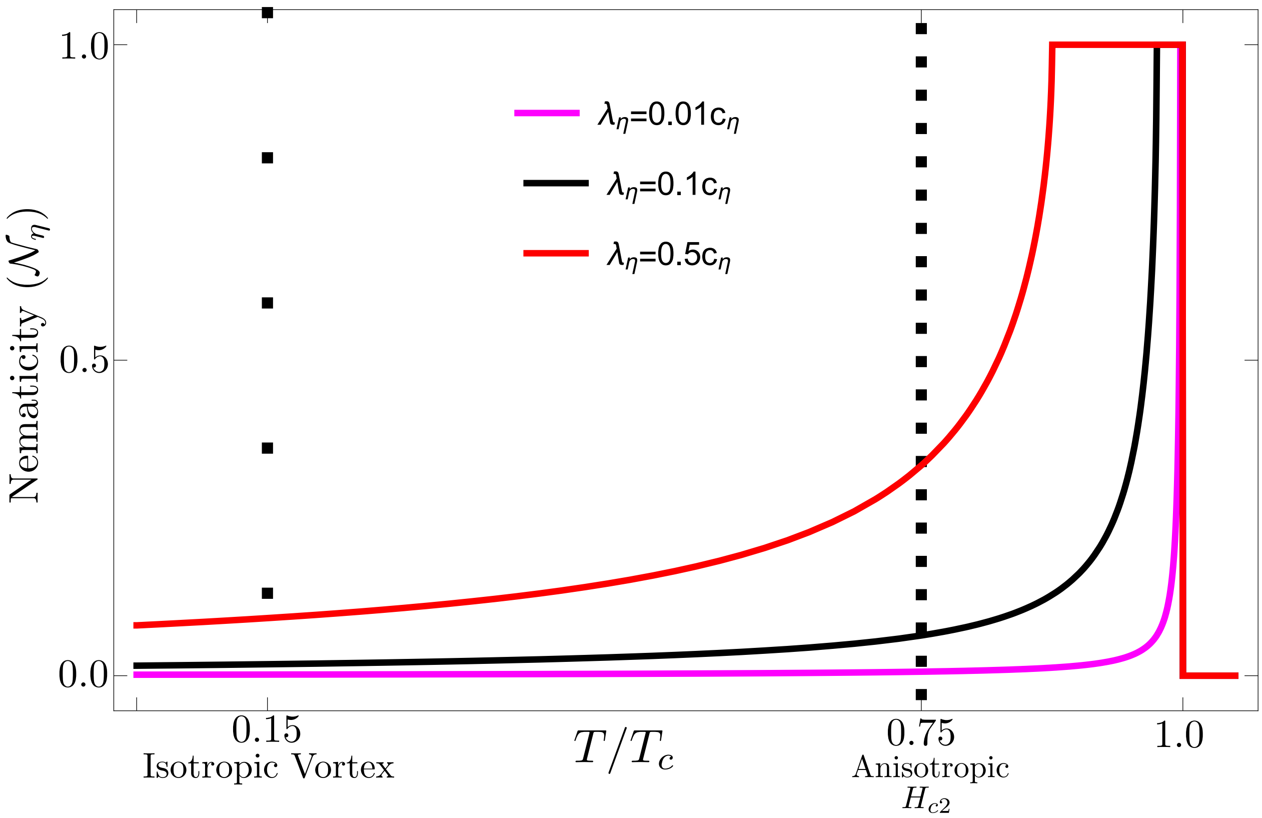

FIG. S12. The variation of degree of anisotropy (nematicity) defined in Eq. (35) as a function of temperature for different coupling strengths. The broken vertical lines shows the approximate temperature regime where different experimental observations are made.

We define a degree of anisotropy, nematicity ($\mathcal{N}_\eta$) as

$$\mathcal{N}_\eta = \frac{\pi/4 - \alpha}{\pi/4}. \tag{35}$$

The dimensionless quantity vanishes when the order parameter is purely chiral and goes to unity when it is purely nematic. Using this quantity, let us compare our findings with the experimental observations, see Fig. S12.

- The experiments have not detected T^* in the specific heat. The theory predicts a kink at T^*. This can be reconciled if T^* is small compared to T_c and hence becomes undetectable. As shown below, other allowed terms in the GL free energy can suppress this feature. Moreover, in the static GL treatment, we did not consider the contribution of the specific heat from the excited quasi-particles that might further diminish any feature at T^*. Allowing a mixture of s-wave order parameter can suppress the kink even further.

- The experiments find anisotropic H_{c2} at least up to $T < 0.75T_c$. The theory predicts nematicity up to $T \sim 0.5T_c$ for $\lambda_\eta > 0.03c_\eta$.

- The experiments detected isotropic vortices at $T \sim 0.15T_c$. The theory predicts the same for $\lambda < 0.2c_\eta$.

This sets the regime of $\lambda \sim 0.1c_\eta$ which sets the $T^* = 0.9T_c$. The minute jump of specific heat at T^* can be hard to detect in experiments. However, the theory predicts, for this parameter regime the vortices should also become anisotropic around $T \sim 0.5T_c$.

IV. Modification to the Free energy – B_z terms

There is another symmetry-allowed term that we have ignored until now. This term is given by,

$$f_B = iB_z(\boldsymbol{\eta}^* \times \boldsymbol{\eta})_z, \tag{36}$$

and it enhances the chiral order. In our representation, this becomes,

$$f_B = -B_z\eta^2 \sin(2\alpha)\sin(\gamma). \tag{37}$$

Let us minimize the total free energy with respect to γ. We obtain

$$-2|c_\eta|\eta^4 \sin^2(2\alpha)\sin(\gamma)\cos(\gamma) - B_z\eta^2 \sin(2\alpha)\cos(\gamma) = 0, \tag{38}$$

which has two solutions, $\gamma = \pi/2$ and $\gamma = \sin^{-1}(-\frac{B_z}{2|c_\eta|\eta^2\sin(2\alpha)})$. The solution $\gamma = \pi/2$ is always the minimum.

Next, we minimize with respect to α and obtain

$$-4|c_\eta|\eta^4 \sin(2\alpha)\cos(2\alpha) + 2\lambda_\eta\eta^2 \sin(2\alpha) - 2B_z\eta^2\cos(2\alpha) = 0. \tag{39}$$

Note that for non-zero B_z, α cannot be zero. We obtain the following transcendental equation

$$\cos(2\alpha) = \frac{2\lambda_\eta b_\eta}{|c_\eta|(T_c - T)a_\eta}\left(1 - \frac{B_z}{\lambda_\eta}\cot(2\alpha)\right) \tag{40}$$

Note that the expression reduces to the previous version if $B_z = 0$. The usual graphical methods can obtain the solution of this equation by plotting LHS and RHS as a function of α. The solution is given by the crossing point of the two curves. α obtained using the procedure is shown in Fig. (S13) for different B_z. The T^*-regime of purely nematic state for $B_z = 0$ vanishes as we increase B_z. Although α reduces near T_c, a purely nematic state is never obtained for $B_z \neq 0$. We work in the regime of the parameters, where $B_z \ll \lambda_\eta \ll c_\eta \ll b_\eta$

Next, we evaluate the specific heat with this additional term. Note that the $f_b \propto \eta^2$, hence it has a linear temperature dependence. Therefore, d^2f_B/dT^2 will vanish for this term, hence the expression for the specific heat remains unchanged as before. However, since B_z modifies $\alpha(T)$, it influences the specific heat. In Fig. (S11c), we have compared the specific heat obtained for $B_z = 0.2\lambda\eta$. The sharp kink at T^* reduces drastically as we increase the B_z term.

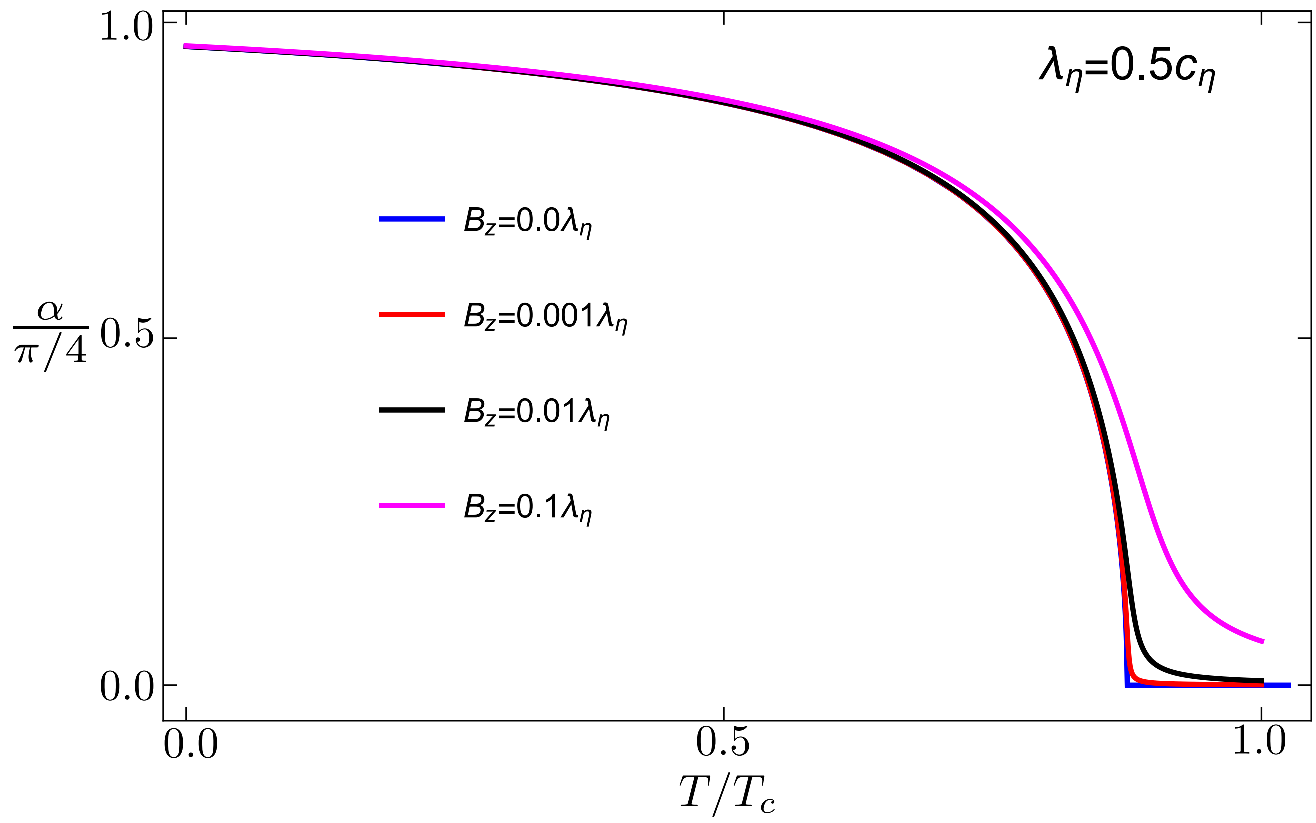

FIG. S13. Variation of the parameter normalized α with temperature for different B_z. The transition at T^* becomes a smooth crossover as the B_z term becomes non-zero.

V. Minimization of the free energy, $\lambda_\eta \ll c_\eta \lesssim b_\eta$

We assume for simplicity that $c_\eta \ll b_\eta$, and therefore obtain $\eta^2 = \frac{a_\eta(T_c - T)}{2b_\eta}$. Next we check the modification of the expressions when we have $\lambda_\eta \ll c_\eta \lesssim b_\eta$. To accomplish this, we again obtain that the for minima $\gamma = \pi/2$. Plugging this into the expression of the free energy, we minimize with respect to α and obtain

$$\alpha = \begin{cases} 0 & T > T^*, \\ \frac{1}{2}\cos^{-1}(\frac{\lambda_\eta}{2c_\eta \eta^2}) & T < T^*. \end{cases}$$

Plugging in $\alpha = 0$ for $T < T^*$, we obtain

$$\eta^2 = \frac{a_\eta(T_c - T)}{2b_\eta} + \frac{\lambda_\eta}{2b_\eta}. \tag{41}$$

T_c slightly increases in this regime, but since $\frac{\lambda_\eta}{2b_\eta} \ll 1$, we can safely ignore such a contribution.

If $\alpha \neq 0$, when we are at $T < T^*$, we obtain the equation for η as

$$2(b_\eta - c_\eta)\eta^2 + r_\eta + \frac{\lambda_\eta^4}{8c_\eta^3\eta^6} = 0. \tag{42}$$

since $\lambda_\eta \ll c_\eta$ and we are far from T_c, we can write

$$\eta^2 \approx \frac{a_\eta(T_c - T)}{2(b_\eta - c_\eta)}. \tag{43}$$

T^* is modified in this regime by

$$T^* = T_c - \frac{(b_\eta - |c_\eta|)\lambda_\eta}{a_\eta |c_\eta|}. \tag{44}$$

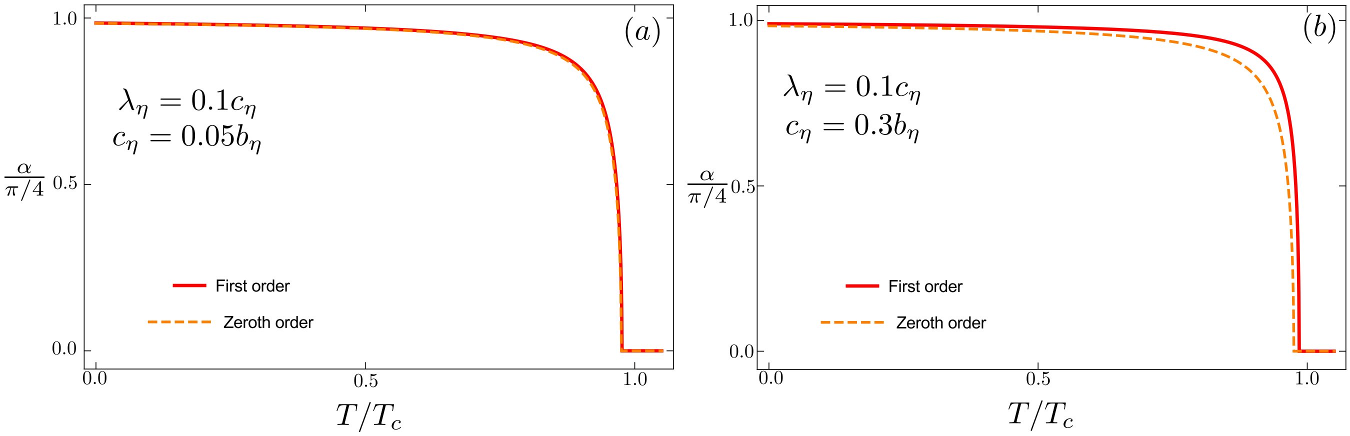

FIG. S14. The variation of the normalized α as a function of temperature for zeroth order approximation $\lambda_\eta \ll c_\eta \ll b_\eta$ and first-order approximation $\lambda_\eta \ll c_\eta \lesssim b_\eta$. (a) For $c_\eta = 0.05b_\eta$ we have a perfect matching between the two. (b) For $c_\eta = 0.3b_\eta$ we have a small deviation.

We have compared the two approximations in Fig. S14. As expected, the two expressions match for small c_η/b_η.

The regime of $\lambda_\eta \lesssim c_\eta \lesssim b_\eta$ is not very interesting, as there the nematicity can survive down to zero temperature, which is in contradiction to the experiment. For completeness, we can comment that for large λ_η, T_c is expected to increase.

S9. Supplementary References

[1] Inada, R., Onuki, Y. & Tanuma, S. Hall effect of 1T-TaS_2 and 1T-TaSe_2. *Physica B+C* **99**, 188–192 (1980).

[2] Thompson, A. H., Gamble, F. R. & Koehler, R. F. Effects of intercalation on electron transport in tantalum disulfide. *Physical Review B* **5**, 2811–2816 (1972).

[3] Ribak, A. *et al.* Chiral superconductivity in the alternate stacking compound 4Hb-TaS_2. *Science Advances* **6**, 9480–9507 (2020).

[4] Conroy, L. E. & Pisharody, K. R. The preparation and properties of single crystals of the 1s and 2s polymorphs of tantalum disulfide. *Journal of Solid State Chemistry* **4**, 345–350 (1972).

[5] Narayan, J. Coexistence of two charge density waves of different symmetry in transition metal dichalcogenide 4Hb-TaS_2. *Applied Physics Letters* **29**, 223–224 (1976).

[6] Scholz, G. A., Singh, O., Frindt, R. F. & Curzon, A. E. Charge density wave commensurability in 2H-TaS_2 and Ag_xTaS_2. *Solid State Communications* **44**, 1455–1459 (1982).

[7] Ravnik, J. *et al.* Strain-induced metastable topological networks in laser-fabricated TaS_2 polytype heterostructures for nanoscale devices. *ACS Applied Nano Materials* **2**, 3743–3751 (2019).

[8] Burk, B., Thomson, R. E., Clarke, J. & Zettl, A. Surface and bulk charge density wave structure in 1T-TaS_2. *Science* **257**, 362–364 (1992).

[9] Varga, K. & Driscoll, J. A. *Computational nanoscience: Applications for molecules, clusters, and solids* (Cambridge University Press, 2011).

[10] Venderbos, J. W. F., Kozii, V. & Fu, L. Identification of nematic superconductivity from the upper critical field. *Physical Review B* **94**, 94522 (2016).

[11] Etter, S. B., Bouhon, A. & Sigrist, M. Spontaneous surface flux pattern in chiral p-wave superconductors. *Physical Review B* **97**, 064510 (2018).
